# Computed Tomography, Coronary Angiography, and Intravascular Ultrasound in the Diagnosis of Left Anterior Descending Stenosis in a 38-Year-Old Woman with a Calcium Score of Zero

**DOI:** 10.3390/diagnostics15091169

**Published:** 2025-05-04

**Authors:** Malgorzata Zalewska-Adamiec, Slawomir Dobrzycki

**Affiliations:** Department of Invasive Cardiology, Medical University of Bialystok, 15-089 Bialystok, Poland

**Keywords:** coronary computed tomography angiography, coronary angiography, intravascular ultrasound, coronary artery calcium score

## Abstract

Cardiovascular diseases, including coronary artery disease, are the leading cause of mortality in the general population. The likelihood of significant coronary artery disease occurring in young women (<40 years of age) is very low. Diagnosis is challenging and often delayed, treatment tends to be suboptimal, and the long-term prognosis is poor. We present the case of a 38-year-old woman with typical anginal chest pain whose coronary computed tomography angiography (CCTA) revealed significant narrowing in the left anterior descending artery (LAD) despite a coronary artery calcium score (CACS) of zero. To assess the significance of the lesion, coronary angiography and intravascular ultrasound (IVUS) were performed, which revealed borderline narrowing. Conservative treatment was recommended.

**Figure 1:**
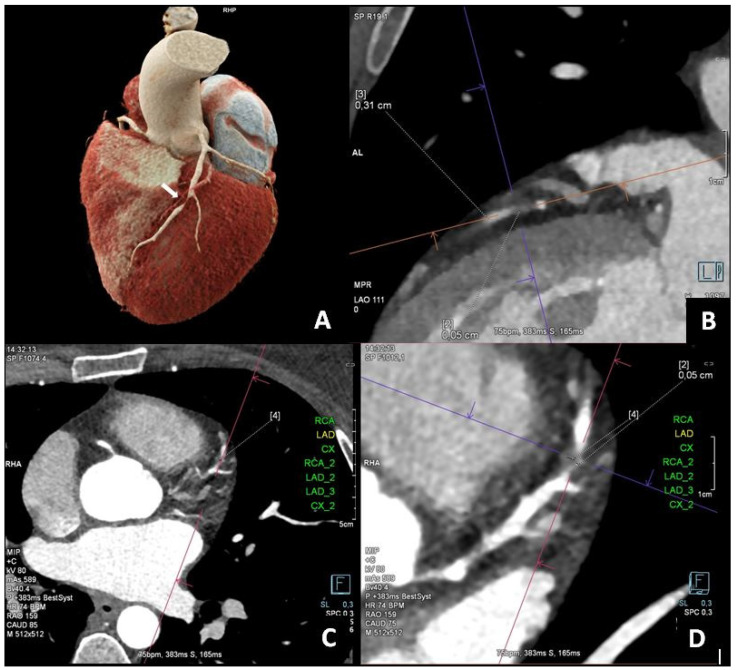
A 38-year-old woman who was diagnosed with hypercholesterolemia, was a former smoker, and had a history of typical exertional angina for about six months was admitted to the clinic for coronary angiography. No ischemic abnormalities were detected in the baseline ECG recordings. An exercise stress test performed on an outpatient basis was normal at a workload of 10 METs. A coronary computed tomography angiography (CCTA) was performed, revealing a coronary artery calcium score (CACS) of 0 according to the Agatston scale. In the mid-segment of the LAD (left anterior descending), a non-calcified, hypodense atherosclerotic plaque measuring up to 10 mm was visualized, causing significant/critical stenosis of approximately 80–90% ((**A**–**D**), white arrow). Features of positive vessel remodeling were observed at the plaque level. No stenoses were found in the proximal or distal segments of the LAD or the remaining coronary arteries.

**Figure 2:**
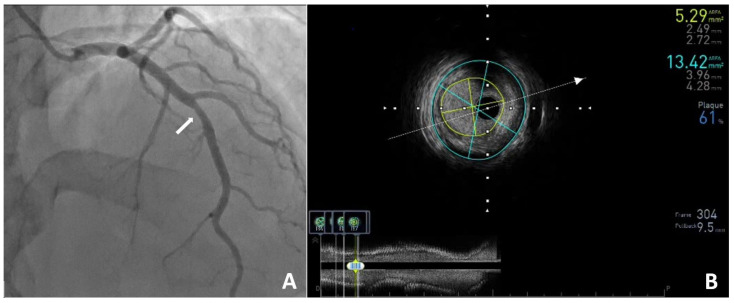
In the clinic, a transthoracic echocardiogram was performed and showed normal valve function and left ventricular contractility with an ejection fraction of 60%. Subsequently, coronary angiography revealed a 50% stenosis in the LAD ((**A**), white arrow), with no other changes in the remaining coronary arteries. The angiographic findings were further evaluated using IVUS (intravascular ultrasound), which showed borderline stenosis in the LAD with the following measurements: MLA (minimal lumen area)—5.29 mm^2^; CSA (cross-sectional area)—13.42 mm^2^; and PB (plaque burden)—61% (**B**). Laboratory investigations performed during hospitalization were within normal limits. Results included the following: total cholesterol—104 mg/dL, LDL cholesterol—43 mg/dL, HDL cholesterol—52 mg/dL, triglycerides—22 mg/dL, lipoprotein(a)—7.7 mg/dL, HbA1c—4.7%, and NT–proBNP—67 pg/mL. No elevation in inflammatory markers was observed. The patient was qualified for conservative management, with pharmacotherapy recommendations including continued use of acetylsalicylic acid (75 mg/day) and atorvastatin (40 mg/day). Nebivolol, which was used previously, was replaced with amlodipine (2.5 mg/day) and trimetazidine (35 mg twice daily) due to a possible vasospastic component. Three months later, the patient returned for a follow-up visit at the cardiology outpatient clinic. She reported feeling well and denied any substernal chest pain. Continuation of the current treatment regimen and regular cardiology follow-up were recommended. In our patient, the probability of significant coronary artery disease was very low [1]. As part of the initial diagnostic evaluation, the patient underwent a stress test. However, in this group of patients, an ischemia imaging test, such as stress cardiac magnetic resonance (CMR) or stress echocardiography, could have been considered [2]. The CACS in our patient was 0, whereas CCTA revealed a lesion in the proximal segment that required further evaluation via coronary angiography and intravascular ultrasound [3]. This case illustrates that, in young individuals, a zero CACS does not exclude the presence of significant coronary artery disease [4,5,6,7]. Therefore, in this population, a personalized diagnostic approach should be applied, taking into account risk factors and clinical symptoms.

## Data Availability

Original data supporting the reported results are available by contacting the authors.
